# Characteristics and mechanisms of liver injury caused by emerging infectious diseases

**DOI:** 10.3389/fimmu.2025.1647517

**Published:** 2025-10-15

**Authors:** Yi Cheng, Xin Zheng

**Affiliations:** Department of Infectious Diseases, Union Hospital, Tongji Medical College, Huazhong University of Science and Technology, Wuhan, China

**Keywords:** liver injury, COVID-19, dengue, SFTS, Ebola, mitochondrial damage, endoplasmic reticulum stress, apoptosis

## Abstract

Abnormal liver function has become a common phenomenon in emerging infectious diseases caused by viruses, with incidence rates ranging from 2.5% to 98.6% across different pathogens. This review summarized the characteristics of liver injury caused by SARS-CoV-2, MERS-CoV, H7N9, SFTSV, DENV, and EBOV viruses. Viral infection initiates liver injury through direct attack, ischemia, and microthrombosis, triggering an exaggerated immune response often exacerbated by drug toxicity. Core mechanisms involve interconnected mitochondrial dysfunction (causing energy failure, ROS/mt-DNA release), endoplasmic reticulum stress (with dual roles in adaptation and apoptosis), and aberrant inflammation. These pathways form a vicious cycle, culminating in hepatocyte death, metabolic disruption, and severe hepatic damage. An in-depth exploration of the causes and mechanisms of liver injury also provides diversified strategies for treating and preventing these infectious diseases.

## 1 Introduction

Emerging infectious diseases are defined as rapidly spreading contagious diseases caused by new or existing but not widely recognized pathogens. In the past 30 years, a variety of emerging infectious diseases have emerged worldwide, including Corona Virus Disease 2019 (COVID-19), Middle East Respiratory Syndrome (MERS), H7N9 avian influenza, dengue fever (Dengue), severe fever with thrombocytopenia syndrome (SFTS) and Ebola, which pose a serious threat to human health.Sars-CoV-2, MERS-CoV, H7N9, DENV (Dengue Virus), SFTSV (Severe Fever with Thrombocytopenia Syndrome Bunyavirus), EBOV (Ebola virus) infection can cause flu-like symptoms, including fever, fatigue, dyspnea, and other symptoms. In addition to flu-like symptoms, these infections share a common feature—liver injury, which plays a critical role in disease progression. Studies have shown that liver injury is highly prevalent in emerging infectious diseases, with the incidence of abnormal liver function in 2.5%-96.8% (1), 31.4% (2), 29% (3), 60-90% (4), 96.6-98.6% (5, 6) and >70% (7) of COVID-19, MERS, H7N9 avian influenza, dengue fever, SFTS, and Ebola patients, respectively. This suggests that liver injury plays a vital role in emerging infectious diseases. However, the mechanisms by which these infectious diseases cause liver injury and their relationship to disease progression remain controversial. However, a comparative and integrative understanding of how these diverse pathogens converge on common host pathways to drive immunopathological liver injury remains lacking. This review aims to fill this critical knowledge gap by proposing a unified framework centered on key mechanisms including mitochondrial dysfunction, ER stress, and immune imbalance.

This review focuses on emerging and re-emerging viruses—including SARS-CoV-2, MERS-CoV, H7N9 influenza, dengue, and Ebola—selected for their significant public health threat, well-documented but diverse liver injury patterns, and sufficient mechanistic literature. We excluded viruses such as yellow fever (YFV) and hepatitis E (HEV), whose hepatic involvement is a primary feature, to better analyze indirect and extra-hepatic mechanisms of liver dysfunction in systemic infections.

## 2 Characteristics of liver injury in emerging infectious diseases

Although liver injury is a common feature after SARS-CoV-2, MERS-CoV, H7N9 avian influenza, DENV, SFTSV, and EBOV infection, the characteristics of liver injury caused by different viral infections vary. The clinical and pathological characteristics of liver injury caused by the above six emerging infectious diseases are summarized in **Tables 1** and **2**.

**Table 1:** Clinical characteristics of liver injury caused by emerging infectious diseases.

Virus	Disease	Incidence of liver injury	Manifestations of liver injury	Relationship to severity and poor prognosis
SARS-CoV-2	Corona Virus Disease 2019 (COVID-19)	2.5–96.8% (1)	ALT (20-80.22%), AST (3.1-52.1%), TBIL (23-54%), γ-GGT (1-58.5%), ALP (54.78%) increased and ALB decreased (1, 67).	ALT, AST, ALP, DBIL, CRP, ferritin, IL-6, -10, and ALB were independent indicators of severity and mortality (1).
MERS-CoV	Middle East Respiratory Syndrome (MESR)	31.4% (2)	Increased ALT (11-33.2%), AST (15-67.1%), LDH (47-88.4%), BIL (12.4%) and decreased ALB (49.3%) levels (68, 69).	Elevated transaminases were significantly associated with disease severity and high risk of mortality (68). Decreased ALB levels are an independent risk factor for severe infections and intensive care (2).
H7N9 avian influenza virus	H7N9 avian influenza	29% (3)	Increased ALT, AST, TBIL, and LDH levels (70). The incidence of ALT elevations > 20 × ULN was 1.8% (3).	ALT levels correlate with disease severity. Elevated AST and LDH, on the other hand, are associated with a risk of death (3).
DENV	dengue fever (Dengue)	60-90% (4)	ALT (45-96%), AST (63-97%), ALP (19.1%) increased, hyperbilirubinemia (12-48%), hypoalbuminemia (12.9-67%), INR > 1.5 (11%) (71, 72).	Elevated transaminases are associated with disease severity, and ALT, AST, INR, bilirubin, ALP, ALB are predictors of acute liver failure or death (72, 73). INR ≥ 1.5 is associated with a high risk of mortality in dengue (73).
SFTSV	severe fever with thrombocytopenia syndrome (SFTS)	96.6-98.6% (5, 6)	Elevated LDH (98.8%), AST (93.5-98.4%), and ALT (79.0-90.4%) levels (5, 74).	Patients with ALT > 1 ULN, ALP > 2 ULN, GGT > 2 ULN, and ALB < 30 g/L are associated with an increased risk of death (6).
EBOV	Ebola	——	ALT or AST > 5 ULN in 70% of patients, and AST > 15 ULN occurred in 44% of non-fatal cases and increased to 93% of fatal cases (7).	ALT, AST, LDH, APTT, and INR levels correlated with viremia levels. ALT, AST, BIL, APTT, and INR were significantly associated with prognosis. High levels of AST were significantly associated with death (75).

**Table 2:** Pathological characteristics of liver injury caused by emerging infectious diseases.

Disease	Pathological characteristics		Reference
	Liver cells	Liver structure	
COVID-19	mild steatosis of hepatocytes, mitochondrial swelling, and decreased glycogen granules; punctate and patchy necrosis of hepatocytes	Sinusoidal congestion, mild dilatation, and a small amount of lymphocyte infiltration	(67, 76)
MESR	hepatocyte edema, steatosis	sinusoidal congestion, and hemorrhage; mild lymphocyte infiltration in portal vein and lobules	(77)
H7N9 avian influenza	hepatocyte micro- and macrovesicular steatosis;punctate liver necrosis, mid-lobular cardio-hepatic necrosis without inflammation	sinusoidal congestion, and mild hepatocellular atrophy	(3)
Dengue	Hepatocyte swelling, increased lysosomes, mitochondria swelling. Hepatocyte nuclei were pyknotic, divided, and lysed, with occasional eosinophilic bodies. Hyperplasia of Kupffer cells. Deposition of membrane attack complex C9 and DENV antigens was observed in hepatocytes, Kupffer cells, and macrophages	No obvious infiltration of immune cells.	(78)
SFTS	Hepatocyte steatosis	there were multiple lobular necrosis, mild portal fibrosis, mild periportal lymphocytic infiltration, and focal cholestasis	(79)
Ebola	Hepatocyte dot-like to large patchy necrosis. Mild to moderate steatosis of hepatocytes. Hyperplasia of Kupffer cells. Quite a few viral inclusions were present in hepatocytes and bile ducts	Dilated sinusoids, congestion. Plenty of inflammatory cells infiltrated around the portal vein and necrosis.	(24)

In addition to abnormal liver function, the interaction between concurrent liver diseases and the above infectious diseases has also aroused high concern (**Table 3**). SARS-CoV-2 has been well studied in combination with prior liver disease. Relatively speaking, studies on co-infection of other liver diseases with MERS-CoV, H7N9, and SFTS are limited. There are no systematic studies between Ebola and hepatic complications.

**Table 3:** The interaction between concurrent liver diseases and infectious diseases.

Disease	Concurrent liver diseases			
	Chronic hepatitis B (CHB)	Nonalcoholic fatty liver disease (NAFLD)	Cirrhosis	Hepatoma and liver transplantation
COVID-19	Accompanied by higher levels of ALT, AST, and APTT, a longer viral clearance time, a higher risk of severe disease, and a worse prognosis, but has no significant impact on mortality (80).	Accompanied by longer viral shedding time and higher risk of disease progression, but there was no significant difference in the risk of death (81).	Accompanied by lower risk of SARS-CoV-2 infection, but more likely to develop decompensated, acute-on-chronic liver failure (82). Cirrhosis was an independent predictor of mortality (83).	Accompanied by higher risk of SARS-CoV-2 infection. The mortality rate of liver cancer patients increased with Barcelona stage (84). The mortality rate of liver transplantation patients (17% – 18%) was consistent with the expected mortality rate (85).
MESR	——	——	Positive correlation with mortality rate (86).	——
H7N9 avian influenza	More susceptible to avian influenza virus infection (87).	——	More susceptible to avian influenza virus infection (87).	More susceptible to avian influenza virus infection (87).
Dengue	Increased the risk of ALF in dengue patients, but did not increase the risk of death (73).	Increased the risk of ALF in dengue patients, but did not increase the risk of death (73).	Accompanied by longer hospital stay and significantly increased 30-day mortality (88).	——
SFTS	No significant differences in the severity of liver injury, indicators of liver abnormalities, and mortality (6).	——	——	——

## 3 Primary causes of liver injury caused by emerging infectious diseases

**Figure 1:**
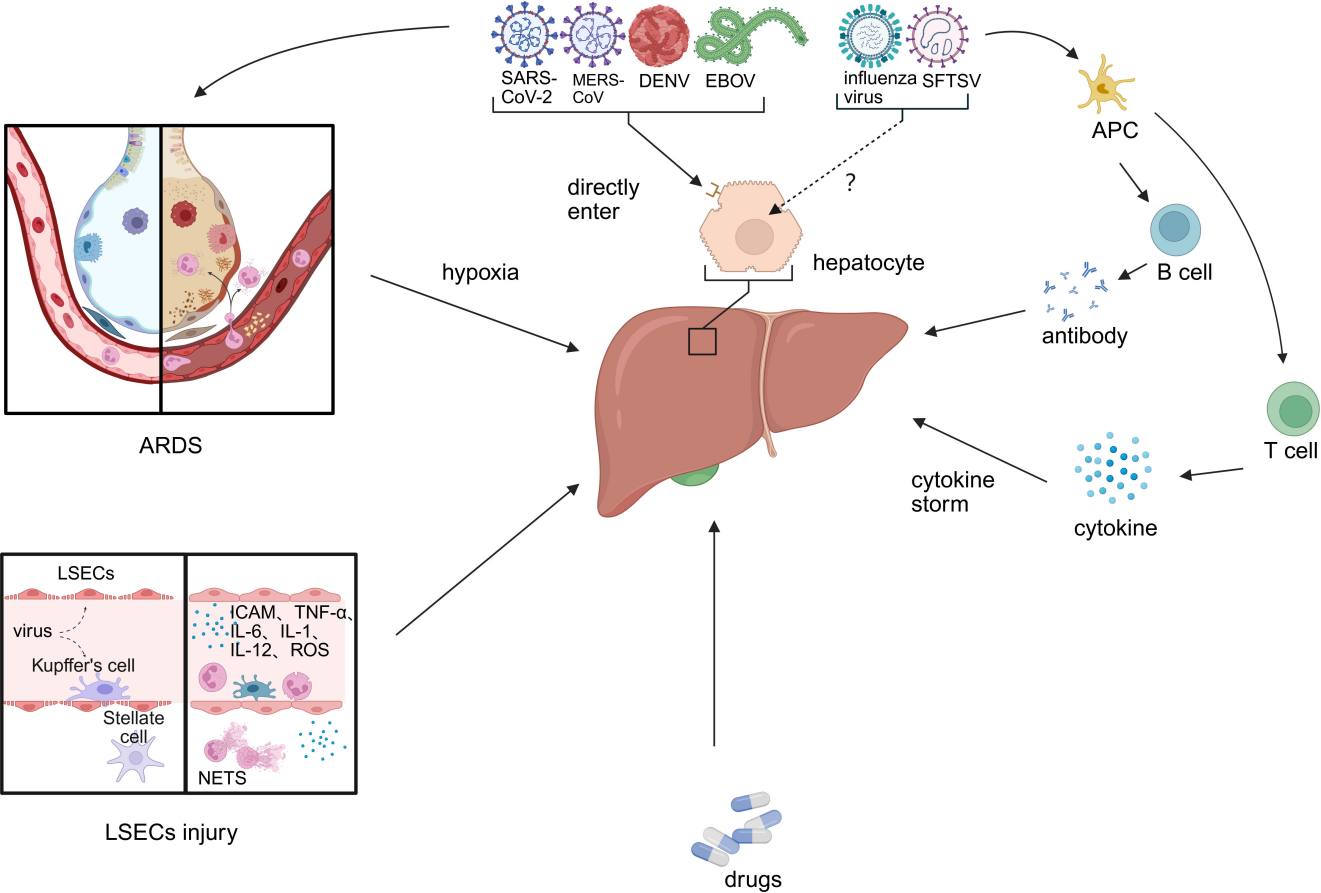
Primary causes of liver injury caused by emerging infectious disease. Viral hepatitis-related liver injury involves multiple interconnected mechanisms, including: (1) direct hepatotropic effects of the virus; (2) ischemia–hypoxia and reperfusion injury; (3) sinusoidal endothelial cell injury and systemic hypercoagulability; (4) immune-mediated damage via inflammatory mediators; and (5) drug-induced liver injury. (LESC, liver sinusoidal endothelial cell; ARDS, acute respiratory distress syndrome; APC, antigen-presenting cell).

The primary causes of liver injury converge into a coherent narrative of pathogenesis (**Figure 1**). The initial direct viral attack on hepatocytes and the endothelial damage that disrupts microcirculation create the foundation for injury. This is dramatically amplified by systemic processes—namely hypoxia and a hypercoagulable state—and by the host’s own robust counterattack through inflammatory mediators. Finally, drug-induced injury often represents a compounding iatrogenic factor. It is the interplay between these direct, indirect, and iatrogenic mechanisms that dictates the ultimate severity of hepatic dysfunction.

### 3.1 Hepatotropic effects of the virus

SARS-CoV-2 binds to ACE-2 via its S protein and enters host cells in concert with TMPRSS2 and FURIN (8). However, few hepatocytes express both ACE2 and TMPRSS2 and no viral inclusion bodies were observed in liver biopsies from COVID-19 patients (9). It has, therefore, been speculated that liver injury in COVID-19 patients may be caused by cholangiocyte injury rather than directly by hepatocytes. ACE2, TMPRSS2, and FURIN expression in hepatocytes are also variable under different disease conditions. Cells expressing ACE2 and TMPRSS2 in the liver were significantly increased in cirrhotic patients, uninfected obese nonalcoholic steatohepatitis patients, and liver transplant recipients and donors, but significantly decreased in untreated HBV-infected patients (10). Taken together, SARS-CoV-2 itself may not directly contribute to liver injury but is associated with increased susceptibility of hepatocytes under pathological conditions.

Unlike SARS-CoV-2, MERS-CoV uses dipeptidyl peptidase-4 (DPP-4) as its functional receptor to enter cells and cause infection. DPP-4 is expressed at higher levels in the liver; therefore, the liver may be its target organ. A transgenic mouse model expressing codon-optimized human DPP-4 (hDPP4) was constructed, and MERS-CoV was observed to invade hepatocytes and cause hepatocyte injury through DPP-4 (11).

DENV enters cells by binding to receptors on the surface of hepatocytes and Kupffer cells via membrane protein E on their surface (12). This process is associated with the serotype of the virus, with DEN1 passing through a 37/67-kDa high-affinity laminin (13), while DEN2 enters hepatocytes via GRP78 (14).

EBOV binds to cell surface-specific receptors via its surface glycoprotein (GP) to guide virions into cells, and vesicles formed by endocytosis are transported to endosomes (15). GP interacts with intracellular receptors in endosomes to promote viral entry and complete host cell invasion (16).

H7N9 avian influenza virus can invade cells by binding SAα-2, 3-GAL and SAα-2, 6-GAL receptors. Among them, α-2,6 SA receptors are mainly expressed in ciliated cells of the human upper respiratory tract. In contrast, α-2,3 SA receptors are primarily expressed in non-ciliated cells and type II pneumocytes of the lower respiratory tract (17). Using CRISPR-Cas9 technology, chemokine receptor 2 (CCR2) was identified as a receptor for SFTSV entry into host cells (18). However, CCR2 expression is mainly restricted to bone marrow, hematogenous cells, and secondary lymphoid organs and is not expressed in hepatocytes (19). Therefore, H7N9 and SFTSV may not be hepatotropic, and the liver injury they cause may be associated with secondary injury. In addition, these two viruses may enter hepatocytes through other receptors and require further experimental studies.

### 3.2 Ischemia-hypoxia and reperfusion injury

The liver has a dual blood supply to the portal vein and hepatic artery. It has a specific buffering capacity for hypoxia. Still, when the degree of hypoxia exceeds its regulatory range, hepatocytes suffer acute hypoxic injury (also known as hypoxic hepatitis), which can be caused by a variety of causes, more than 90% of which are caused by respiratory failure, sepsis, and heart failure. First, patients with COVID-19, MERS, H7N9 avian influenza, SFTS, dengue fever, and Ebola can show varying degrees of hypoxemia, and ARDS is also common in critically ill patients. Second, secondary infection is a common complication in critically ill patients. In the early stage of sepsis, increased hepatic oxygen demand and decreased oxygen utilization are some of the causes of hypoxic hepatitis. Sepsis develops and causes hemodynamic changes, leading to shock. In addition, viruses such as SARS-CoV-2, DENV, and EBOV can directly damage sinusoidal endothelial cells and aggravate tissue hypoxia.

Most early changes in hypoxic hepatocytes occur in mitochondria. Hypoxia immediately interrupts electron transport in the respiratory chain, rapidly consumes ATP, accelerates glycolysis, increases lactate formation, changes in H+, Na+, and Ca2+ homeostasis, increases ROS production, and damages hepatocytes. Subsequently, lipid accumulation, glycogen depletion, and adenosine triphosphate depletion in hepatocytes can inhibit cell survival signaling and rapidly lead to hepatocyte death. Reperfusion following ischemia also exacerbates metabolic disturbances: In the early reperfusion phase, Kupffer cells rapidly activate and release ROS, inducing oxidative stress and vascular injury (20). ROS and their peroxidation products produced during ischemia and reperfusion injury can activate redox-sensitive transcription factors, release various pro-inflammatory factors, and induce liver injury. In the late phase, neutrophils accumulate in the liver after reperfusion and cause damage to hepatocytes through oxidants and proteases (20).

### 3.3 Liver sinusoidal endothelial damage and hypercoagulable state

Endothelial dysfunction is a common consequence of multiple viral infections and one of the causes of liver injury. ACE2 and TMPRSS2 are expressed in sinusoidal endothelial cells, and SARS-CoV-2 has been shown to infect endothelial cells and induce diffuse endothelial inflammation (21). SFTSV can target endothelial cells *in vitro*, disrupt their intercellular junctions, and trigger inflammatory responses, increasing permeability (22). Vacuolization following sinusoidal endothelial cell injury has been observed in DENV-infected mouse models (23). In ultrastructural analysis of fatal cases of EBOV infection, numerous inclusions and viral particles were found within endothelial cells (24). Although there are no reports on sinusoidal endothelial injury directly caused by MERS and H7N9 avian influenza, based on the fact that severe hypoxia can lead to sinusoidal endothelial injury, it can be inferred that sinusoidal endothelial injury also exists in MERS and H7N9 avian influenza.

In the injured area, endothelial cells change from anticoagulant to procoagulant phenotype, release vWF, bind to GPIb/IX complex on the platelet surface, and promote platelet adhesion, aggregation, and formation of a large number of platelet thrombi to the injured site. After platelets adhere to the injured site, they release fibrinogen, VWF, PAI, TXA2, coagulation factors, etc., while endothelial cells release ADP, PAF and other substances to promote platelet activation and aggregation. Thus, procoagulant activity increases after endothelial injury, anticoagulant substances decrease, and the fibrinolytic system is relatively inhibited, which induces a hypercoagulable state. In addition, the development of a hypercoagulable state is also associated with immunity. High levels of IL-6 can induce megakaryopoiesis and platelet formation. In the later stages of hepatic ischemia-reperfusion injury, many neutrophils accumulate, releasing neutrophil traps and activating platelets.

### 3.4 Immune damage caused by inflammatory mediators

Cytokine storm is a severe clinical phenomenon in which immune cells proliferate excessively and inflammatory cytokines are released in large amounts, resulting in multiple organ system failures and tissue damage. Although cytokine storm caused by different viral infections has unique characteristics, IFN-γ, IL-1, IL-6, TNF, and IL-18 are critical to the elevation in cytokine storms and are thought to have central immunopathologic effects (25).

First, cytokines can damage hepatocytes through local effects. Cytokine storm activates Kupffer cells even in the absence of viral antigens in the liver (1), which, upon activation, release cytokines, ROS, and NO, inducing sinusoidal endothelial injury and perpetuating hepatocellular injury (26). TNF-α can directly stimulate hepatocytes to produce IL-6, promote the production of caspase-3, and induce hepatocyte apoptosis. Typically, IL-6 has the effects of protecting the liver and regulating liver regeneration; however, high levels of IL-6 disturb liver regeneration, stimulate hepatocytes to produce various pro-inflammatory factors, promote hepatocyte steatosis, and exacerbate sinusoidal endothelial cell injury (27). Following sinusoidal endothelial injury, leukocytes can be recruited by expressing adhesion molecules such as ICAM1, further increasing the release of inflammatory factors (28). NO produced by neutrophil activation can induce the formation of peroxynitrite (potent ROS), damage mitochondria, and promote hepatocyte necrosis and apoptosis. Second, cytokines can trigger vascular dysfunction so that the liver is in a state of hypoperfusion. TNF-α can alter endothelial cell morphology and affect NO-mediated vasodilation of the vascular bed (26). The interaction between sinusoidal endothelial cells, Kupffer cells, and leukocytes following injury leads to the redistribution of intrahepatic blood flow, resulting in decreased sinusoidal perfusion. Finally, cytokines can also cause liver damage via the renin-angiotensin-aldosterone system. The S protein of SARS-CoV-2 binds ACE2 and causes a significant increase in Ang II levels in serum, which in turn mediates trans-signaling of the IL-6/sIL-6 receptor complex, and ultimately upregulates the expression of a variety of inflammation-related genes, including NF-κB (29). While Ang II levels increase, Ang (1–7) levels decrease, while Ang (1–7) can down-regulate the expression of p38 MAPK and NF-κB, has anti-proliferative, anti-thrombotic and anti-inflammatory functions, and can improve tissue injury (29). In summary, cytokines can damage the liver through local effects, changes in vascular function, and the RAAS system.

### 3.5 Drug-induced injury

Antibiotics, antivirals, antipyretic and analgesic drugs, and steroids are widely used to treat emerging infectious diseases, all potential factors for liver injury. To date, antiviral drugs widely used to treat the above contagious diseases include ribavirin, favipiravir, lopinavir/ritonavir, chloroquine, interferon, and monoclonal antibodies, and their characteristics and mechanisms of causing liver injury are shown in **Table 4**.

**Table 4:** Characteristics and mechanism of liver injury induced by drugs for infectious diseases.

Drugs		Indications	Characteristics of liver injury	Mechanism of liver injury
Nucleotide and Nucleoside Analogue Inhibitors	Radexivir	Paramyxoviruses, Filoviruses, and Coronaviruses infection	Transaminases increased (89).	Inhibition of human mitochondrial RNA polymerase, induction of reactive oxygen species production (90).
	Farpiravir	SARS-CoV-2, H7N9, SFTSV and EBOV infection	Transaminases increased, cholestasis (rare) (91).	Mt-DNA damaged (92).
	Ribavirin	SARS-CoV-2, H7N9 and SFTSV infection	Not associated with significant liver injury (93).	Cause a dose dependent red cell hemolysis (93).
HIV protease inhibitors	Lopinavir/ritonavir	HIV, SARA-CoV2 and MERS-CoV infection.	AST, ALT and TB increased (94).	It disrupts the integrity of cell membranes, affects the function of mitochondria and endoplasmic reticulum, and exacerbates oxidative stress (95).
IL-6 inhibitor	Tocilizumab	COVID-19 patients with high inflammatory response.	Transient mild transaminase elevations (96).	Blocks IL-6 pathway associated with liver regeneration (97).
JAK/STAT inhibitors	Baritinib	COVID-19 patients with high inflammatory response.	Transient mild transaminase elevations (98).	CYP3A4 related (99).
Immunomodulators	Interferon	SARS-CoV-2, MERS-CoV and EBOV infection.	Elevated transaminases and autoimmune hepatitis (100).	Activate inflammatory pathways (100).
	Hydroxychloroquine and chloroquine	SARS-CoV-2 and DENV infection.	Rarely.	Its metabolites accumulate in the liver (101).
	Glucocorticoid	Patients with severe COVID-19; H7N9, SFTSV and DENV infection.	Hepatic steatosis and glycogen degeneration (102).	Affects the metabolism of triglycerides and glucose (102).
non-steroidal anti-inflammatory drugs(NSAIDs)	Acetaminophen	Virus-induced fever.	Excessive intake can induce severe liver damage or failure (103).	Induction of mitochondrial oxidative stress and dysfunction, depletion of GSH (104).

## 4 Key mechanisms of liver injury caused by emerging infectious diseases

**Figure 2:**
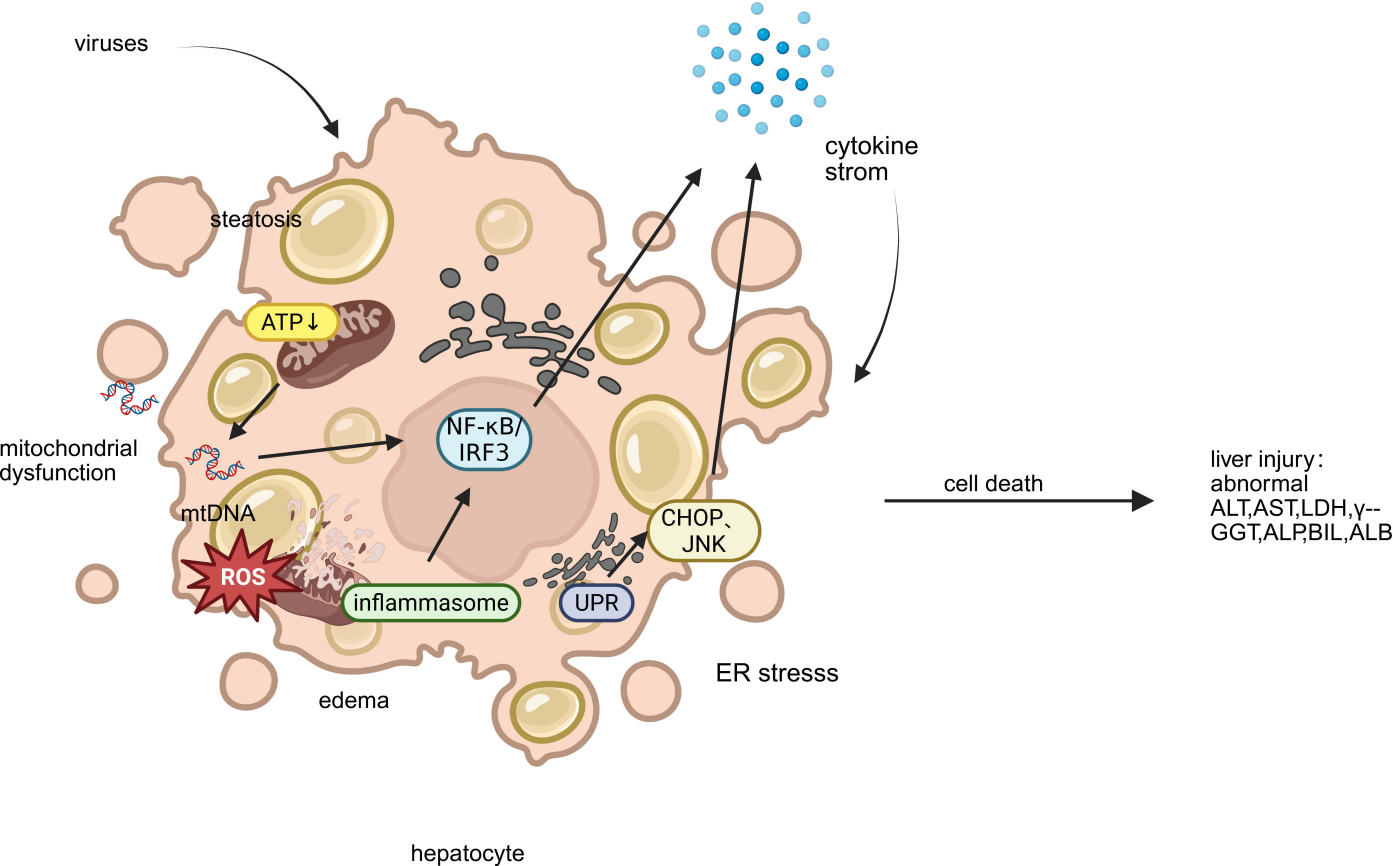
Key mechanisms of liver injury caused by emerging infectious disease. This figure illustrates the key mechanisms of hepatocyte injury triggered by emerging viruses: (1) Mitochondrial Dysfunction, featuring impaired ATP production, ROS release, and mt-DNA leakage; (2) Endoplasmic Reticulum Stress (ERS), showing ER dilatation and the UPR’s dual role in adaptation and apoptosis; and (3) Aberrant Inflammatory Response, where released mt-DNA activates inflammasomes and transcription factors (NF-κB/IRF3), driving a detrimental cytokine storm. These interconnected pathways culminate in cell death, steatosis, and amplified inflammatory liver damage. (ALT, alanine aminotransferase; AST, aspartate aminotransferase; LDH, lactate dehydrogenase; γ-GGT, gamma-glutamyl transferase; TBIL, total bilirubin; ALP, alkaline phosphatase; ALB, albumin).

Recent studies have shown that although different viruses have specific molecular strategies, they can all cause liver cell damage by intervening in mitochondrial function, endoplasmic reticulum homeostasis, and immune response pathways (**Figure 2**). This section systematically summarizes the common mechanisms of mitochondrial dysfunction, endoplasmic reticulum stress (ERS), and abnormal inflammatory response across viruses, and compares the specific regulatory modes of representative viruses in them, providing an integrated mechanism perspective for virus induced liver injury.

### 4.1 Mitochondrial dysfunction

In the pathogenesis of liver damage caused by viruses, mitochondrial dysfunction, as a highly conserved pathophysiological link, constitutes a common molecular basis for various viral pathologies. This mechanism is mainly reflected in three interrelated biological processes: energy metabolism disorders, mitochondrial autophagy mechanism disorders, and abnormal activation of mitochondrial dependent immune signaling pathways. Viral infection affects the function of the electron transport chain, reduces membrane potential, and disrupts the oxidative phosphorylation process, leading to severe damage to ATP synthesis and significant accumulation of reactive oxygen species, thereby triggering oxidative stress. At the same time, the virus intervenes in the mitochondrial quality control system, damaging the mitochondrial autophagy process including PRKN dependent and non-dependent pathways, resulting in the inability to effectively eliminate functionally defective mitochondria. More importantly, the mitochondrial DNA and other molecules released by damaged mitochondria can serve as endogenous danger signals, recognized by cytoplasmic pattern recognition receptors, thereby activating the RIG-I/MAVS signaling axis and downstream NF - κ B and interferon regulatory factor pathways, driving excessive production of inflammatory factors and type I interferons, ultimately exacerbating liver cell damage and death. This series of cascading reactions is not only prevalent in various viral infections, but also highlights the core role of mitochondria in natural immune regulation and maintaining cellular homeostasis.

Viral infection significantly impacts mitochondrial energy metabolism. In SARS-CoV-2, the viral dsRNA contributes to altered mitochondrial membrane potential and ROS generation (30), while its structural protein M impairs electron transport through interactions with host mitochondrial proteins including ACADM, PMPCB, PITRM1, PMPCA, and COQ8B (31). NSP1 of MERS-CoV significantly inhibits the expression of ribosomal and oxidative phosphorylation genes expression, further compromising mitochondrial function (32). DENV infection can induce lipophagy to mobilize fatty acids for mitochondrial β-oxidation and enhance ATP production, promoting viral replication (33). H7N9 (34) and SFTSV (35) infection also leads to loss of membrane potential and oxidative stress. The effect of EBOV infection on mitochondrial energy metabolism has not yet been thoroughly investigated.

Mitophagy, selective autophagy of mitochondria, is a critical mechanism in quality control and rapid turnover of damaged mitochondria. It can be triggered by factors such as hypoxia, mitochondrial depolarization, and viral infection. Mitophagy mechanisms in mammalian cells can be divided into two categories according to the dependence of the E3 ligase PRKN: PRKN-dependent and PRKN-independent (36). PRKN-dependent mitophagy is usually associated with alterations in mitochondrial transmembrane potential, whereas PRKN-independent mitophagy is usually associated with autophagic receptors and LC3 interactions in the outer mitochondrial membrane. Viral infections have evolved sophisticated strategies to disrupt these protective mechanisms. SARS-CoV-2 infection activates the PINK1-Parkin-p62 signaling axis but simultaneously inhibits both PRKN-dependent and independent pathways by preventing the critical p62-LC3 interaction, thereby blocking autophagosome encapsulation of damaged mitochondria (30). Similarly, DENV employs its NS4B protein to suppress DRP1-mediated mitochondrial fission and impair both PRKN-dependent and independent pathways, resulting in accumulated dysfunctional mitochondria (37). Furthermore, DENV infection downregulates nuclear-encoded mitochondrial proteins and suppresses mitochondrial biogenesis, creating an irreversible disruption of cellular homeostasis that ultimately leads to cell death (37).

**Figure 3:**
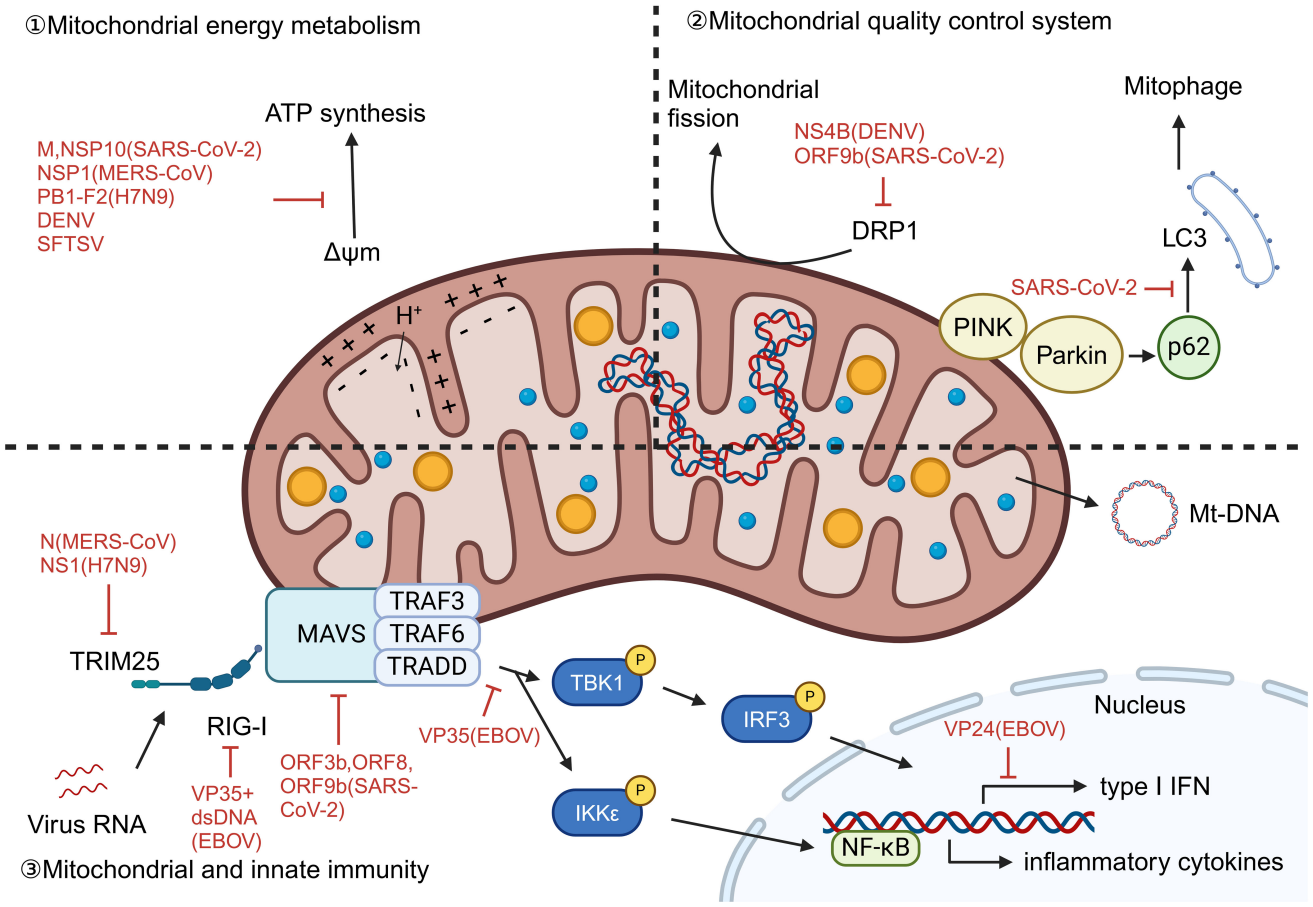
Mitochondrial dysfunction caused by virus infection. Viral infections disrupt mitochondrial function through diverse strategies: (1) Energy metabolism dysregulation: SARS-CoV-2 dsRNA and protein M alter membrane potential and disrupt electron transport; MERS-CoV NSP1 suppresses oxidative phosphorylation genes; DENV induces lipophagy to enhance β-oxidation and ATP production; H7N9 and SFTSV cause membrane depolarization and oxidative stress. (2) Mitophagy disruption: Viruses inhibit mitochondrial quality control—SARS-CoV-2 blocks p62-LC3 interaction impairing both PRKN-dependent and independent pathways; DENV NS4B suppresses DRP1-mediated fission and biogenesis. (3) Innate immune activation and evasion: mt-DNA release triggers RLR/MAVS-mediated IFN and inflammation, but viruses evade immunity: SARS-CoV-2 inhibits IFN-I signaling; MERS-CoV and H7N9 suppress RIG-I/TRIM25; H7N9 PB1-F2 disrupts MAVS complex; EBOV VP35/VP24 block RIG-I sensing and IFN expression; SFTSV uses MAVS-independent IL-1β activation. (ΔΨm, Mitochondrial membrane potential).

Following mitochondrial damage, mt-DNA is released into the cytoplasm and extracellular environment. It acts as damage-associated molecular patterns (DAMPs) by RIG-I-like receptors (RLRs). This triggers the RLR/MAVS signaling cascade, leading to formation of the MAVS signalosome, activation of IRF3 and NF-κB, and subsequent production of type I interferons and proinflammatory cytokines, resulting in antiviral effects. SARS-CoV-2 utilizes *orf3b, orf8, and orf9b* to suppress IFN-I signaling (38). N protein of MERS-CoV (39) and NS1 of H7N9 (40) inhibit RIG-I-induced IFN production by interacting with TRIM25. H7N9 PB1-F2 disrupts MAVS recruitment of TRAF6, TBK1, and IKKϵ (34, 41). EBOV VP35 inhibits IKKϵ and TBK1 activation, binds viral dsRNA to block RIG-I sensing (42), and VP24 suppresses IFN expression downstream of RIG-I/MAVS (43). In contrast, SFTSV evades immunity independently of MAVS, as MAVS deletion does not affect IL-1β induction (35). (**Figure 3**) We explicitly acknowledge that the mechanistic data for EBOV and SFTSV in our study are limited. Although we observe above findings, the exact immune evasion pathways are not fully resolved.

### 4.2 Endoplasmic reticulum stress

The endoplasmic reticulum is a key organelle for protein synthesis and folding, playing a vital role in maintaining cellular homeostasis. As the central organ of metabolism, hepatocytes possess a highly developed endoplasmic reticulum system to support complex functions such as protein synthesis, modification, and lipid metabolism. When hepatocytes are exposed to adverse conditions such as viral infection, hypoxia, or oxidative stress, the accumulation of unfolded and misfolded proteins in the endoplasmic reticulum triggers the unfolded protein response (UPR). This stress response operates through three highly conserved signaling pathways—IRE1α-XBP1, PERK-eIF2α, and ATF6—which work in coordination to enhance the protein-folding capacity of the endoplasmic reticulum and reduce the protein load, thereby restoring cellular homeostasis. In the short term, the UPR alleviates stress through adaptive mechanisms such as upregulating chaperone expression and halting protein translation. However, prolonged or severe ERS shifts toward pro-apoptotic signaling, ultimately leading to hepatocyte death. Multiple viruses can specifically exploit or interfere with different branches of the UPR and downstream endoplasmic reticulum quality control pathways—such as ER-associated degradation (ERAD) and endoplasmic reticulum autophagy—not only creating a favorable intracellular environment for viral replication but also exacerbating metabolic dysregulation and inflammatory responses in hepatocytes, thereby forming a common pathophysiological axis in the development and progression of virus-induced liver injury.

The mechanism of UPR activation by different viral infections is not the same. For example, SARS-CoV-2 activates the UPR via the IRE1α and ATF6 pathways (44), MERS-CoV via the PERK pathway (45), avian influenza virus (46), and EBOV (47) via the IRE1α pathway. Three canonical pathways of the UPR are activated during SFTSV and DENV infection (48, 49).

UPR activation facilitates viral spread and replication and increases the severity of viral infection. *In vitro* studies have found that SARS-CoV-2 can maintain intracellular NUAK2 kinase levels through the IRE1α pathway, allowing the virus to enter cells through the secretion of soluble messengers and enhance viral transmission between cells (50). In addition, serum levels of GRP-78, an alternative receptor for SARS-CoV-2, increase following UPR activation (51), further promoting viral replication. In SFTSV infection, activation of the UPR prolongs the lifespan of infected cells, thereby increasing levels of progeny viruses (48). DENV then regulates BIP expression by binding to the UPR sensor, favoring viral survival and proliferation (49). EBOV hijacks all three protein inhibitory mechanisms in ER phagocytosis and downregulates GP1, 2, increasing viral fitness (52).

**Figure 4:**
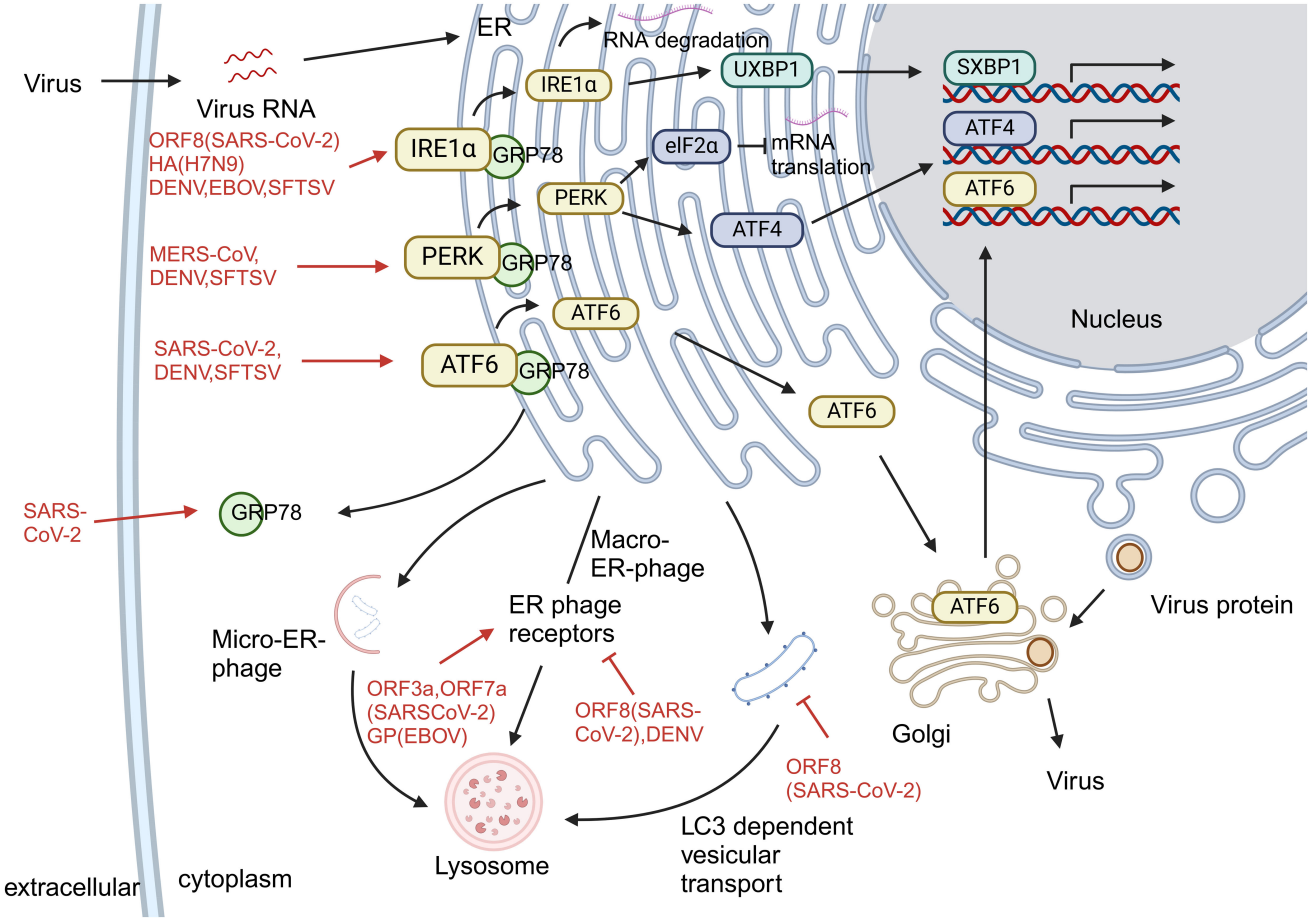
Endoplasmic reticulum stress caused by virus infection. Viral infections differentially activate the UPR and exploit ER quality control pathways to promote replication and evade host defenses. (1) Virus-specific UPR activation: SARS-CoV-2 activates IRE1α and ATF6 pathways; MERS-CoV induces PERK signaling; avian influenza and EBOV trigger the IRE1α pathway; SFTSV and DENV activate all three UPR branches. (2) Subversion of ER quality control: Viruses manipulate ER-associated degradation (ERAD) and autophagy—SARS-CoV-2 ORF3a induces ER-phagy via HMGB1–BECN1, while ORF7a blocks autophagosome-lysosome fusion; DENV cleaves FAM134B via NS2B–NS3 to disrupt ER stability; EBOV utilizes FAM134B-mediated autophagy to degrade GP and VP40. (3) Functional outcome: UPR activation enhances viral replication and spread through various mechanisms, including upregulation of viral entry factors, modulation of chaperone expression, and prolongation of host cell survival.

During endoplasmic reticulum stress (ERS), the unfolded protein response (UPR) not only promotes correct protein folding but also activates two major quality control systems: the ERAD pathway and the autophagy-lysosome system (53). ER, autophagy is another key process to maintain ER quality, involving three pathways: ER macroautophagy, ER microautophagy, and LC3-dependent vesicle trafficking, driving the degradation of intracellular substances such as proteins and membranes (54). SARS-CoV-2 *orf3a* induces ER-phagy via the HMGB1–BECN1 pathway (55), while *orf7a* prevents autophagosome–lysosome fusion by cleaving SNAP29 (56), enhancing viral replication. DENV NS2B–NS3 protease cleaves the ER-phagy receptor FAM134B, disrupting ER stability and promoting viral proliferation (57). The degradation of viral proteins GP and VP40 of EBOV is associated with FAM134B-mediated autophagy processes (58). Although the relationship between MERS-CoV, H7N9, and SFTSV infection and ER autophagy is equally of concern, relevant studies are currently insufficient and require further exploration in the future (**Figure 4**).

### 4.3 Abnormal inflammatory response

During virus-induced liver injury, aberrant inflammatory responses serve as a key shared mechanism, primarily characterized by metabolism dysregulation-induced hepatocyte damage and activation of multiple programmed cell death pathways. Mitochondrial dysfunction and endoplasmic reticulum stress collectively lead to reduced ATP synthesis, disruption of ion homeostasis, and abnormal lipid metabolism, resulting in hepatocyte edema and steatosis. Meanwhile, viruses trigger apoptotic signaling through various mechanisms: on one hand, activating the extrinsic caspase-8 pathway via death receptors, and on the other hand, inducing cytochrome c release and caspase-9 activation through the mitochondrial pathway. Endoplasmic reticulum stress further synergistically promotes apoptosis through signaling molecules such as CHOP, JNK, and caspase-12. Although different viruses may preferentially utilize distinct apoptotic pathways, they ultimately converge on caspase-3 activation, leading to hepatocyte death and further amplification of inflammatory responses, creating a vicious cycle.

Viral infection induces mitochondrial damage in hepatocytes, leading to reduced ATP production and impaired sodium-potassium pump function, which causes hepatocyte edema (32, 38). Additionally, viruses promote intracellular fatty acid accumulation and steatosis through distinct mechanisms: SARS-CoV-2 activates mTOR signaling to enhance *de novo* lipogenesis (59), while DENV‐induced TNF‐α secretion triggers insulin resistance via IRS-1 suppression, promoting gluconeogenesis and lipid accumulation (60). Although inducing host lipogenesis is detrimental to the host, nascent fat can provide the virus with sufficient lipids to produce the vesicular system required for viral replication and exocytosis.

The final stage of aberrant inflammatory response is programmed cell death, or apoptosis, in hepatocytes (61). Hepatocyte apoptosis includes two basic pathways: extrinsic pathway and intrinsic pathway. In hepatocytes, apoptotic signals from death receptors are often insufficient to initiate the caspase cascade, thus generally allowing a mitochondria-mediated pathway to amplify it. SARS-CoV-2 *orf3a* activates caspase-8 and caspase-3 via the extrinsic route, with subsequent mitochondrial cytochrome c release amplifying apoptosis through caspase-9 (62). H7N9 virus activates caspases-8, caspases-9, and caspases-3 in monocytes, and H7N9 may also induce endogenous and exogenous apoptosis in hepatocytes through the exact mechanism (63). SFTSV promotes intrinsic apoptosis through BAK/BAX activation and mt-DNA release (35), while DENV primarily induces intrinsic apoptosis characterized by mitochondrial depolarization and caspase-9/3 activation (64). Limited studies are available on promoting hepatocyte apoptosis by EBOV.

The ER pathway is now of great interest and is regarded as a third apoptotic pathway in parallel with the intrinsic and extrinsic pathways. The UPR in the ERS state is primarily a cell survival mechanism, but apoptosis mediated by crosstalk between the ER and mitochondria is activated during sustained stress. ER stress is mainly involved in apoptosis through four pathways: caspase-12, CHOP, JNK, and Ca2+. For example, *orf3a* of SARS-CoV-2 also promotes caspase-12-mediated ER-specific apoptosis while inducing ER stress (55). In DENV2 infection, CHOP and JNK pathways induce hepatocyte apoptosis; PERK is also involved in mitochondria-associated apoptosis (65). PERK also plays a critical role and is a significant regulator during MERS-CoV infection (66).

## 5 Discussion

Liver injury caused by emerging infectious diseases is usually characterized by elevated transaminase and bilirubin levels and decreased ALB levels, but the specific manifestations vary according to the pathogen. Liver damage is often associated with rapid disease progression and poor prognosis. More severe disease, slower rate of recovery, and worse prognosis are usually observed in patients with concurrent disease in the liver. In terms of liver pathology, hepatocyte necrosis, inflammatory infiltration, and steatosis were prevalent, and ultrastructure showed destruction such as mitochondrial swelling and endoplasmic reticulum deformation.

Liver injury caused by viral infection is a complex process involving multiple factors. It begins with direct infection, leading to ischemia and microvascular thrombosis, which in turn triggers an excessive immune inflammatory response, with potential additional injury conferred by hepatotoxic drugs. Mitochondrial dysfunction, ERS, and aberrant inflammatory responses constitute interconnected core pathological mechanisms in virus-induced liver injury. Mitochondrial damage leads to an energy crisis, ROS accumulation, and mt-DNA release, which in turn activates inflammatory pathways; meanwhile, ERS attempts to restore homeostasis through the UPR, yet prolonged stress shifts toward pro-apoptotic and pro-inflammatory signaling. Ultimately, these intracellular stress signals and the extracellular cytokine storm form a vicious cycle, collectively contributing to hepatocyte death, metabolic dysregulation, and severe inflammatory damage. Furthermore, the impact of pre-existing liver conditions on patient outcomes in SFTS and EBOV, as well as the specific mitochondrial and ER mechanisms underlying hepatotoxicity in these infections, remain poorly understood and represent important avenues for future investigation.

This review has several limitations. First, available research is unevenly distributed, with abundant studies on COVID-19 but far fewer on viruses like EBOV and SFTSV. Second, the lack of standardized criteria for defining liver injury across studies limits direct comparison of results. Finally, mechanistic insights for some pathogens rely heavily on *in vitro* or animal models, which may not fully represent human disease.

Importantly, the delineation of these convergent pathways shifts the therapeutic perspective from solely targeting the virus to also protecting the host. The core mechanisms of mitochondrial dysfunction, ER stress, and aberrant inflammation are highly druggable. Pharmacological modulation of these pathways with organelle-protective or senolytic compounds offers a compelling strategy for mitigating collateral liver damage, potentially improving patient outcomes across diverse viral infections.

This review establishes that viral hepatic injury is a self-perpetuating cycle of organelle stress and inflammatory amplification, thereby advocating for therapeutic strategies that target these convergent host pathways to mitigate collateral damage and improve outcomes.
